# Impact of switching to a dolutegravir‐based regimen on body weight changes: insights from West African adult HIV cohorts

**DOI:** 10.1002/jia2.26371

**Published:** 2024-11-27

**Authors:** Thierry Tiendrebeogo, Karen Malateste, Armel Poda, Albert Minga, Cecile D. Lahiri, Oliver Ezechi, Didier K. Ekouevi, Igho Ofotokun, Antoine Jaquet, the IeDEA West Africa Collaboration

**Affiliations:** ^1^ University of Bordeaux, National Institute for Health and Medical Research (INSERM) UMR 1219, Research Institute for Sustainable Development (IRD) EMR 271, Bordeaux Population Health Research Centre Bordeaux France; ^2^ Department of Infectious Diseases Université Nazi Boni Bobo‐Dioulasso Burkina Faso; ^3^ Centre médical de Suivi des Donneurs de Sang (CMSDS), Centre National de Transfusion Sanguine Côte d'Ivoire (CNTSCI) Abidjan Côte d'Ivoire; ^4^ Division of Infectious Diseases Department of Medicine Emory University School of Medicine Atlanta Georgia USA; ^5^ Office of the Central Secretariat Nigeria Institute for Medical Research Lagos Nigeria; ^6^ Département de Santé Publique Université de Lomé Lomé Togo

**Keywords:** HIV, antiretroviral therapy, dolutegravir, weight gain, observational cohort study, West Africa

## Abstract

**Introduction:**

Adverse metabolic effects related to dolutegravir (DTG) are increasingly reported as countries are adopting DTG‐based regimens as first‐line antiretroviral therapy (ART), but there is limited data from sub‐Saharan Africa. We explored changes in body weight pre‐ and post‐switch to a DTG‐based regimen and assessed the association between DTG switch and significant weight gain (SWG) defined as a ≥10% increase over a 12‐month period in people living with HIV (PLHIV) on ART in West Africa.

**Methods:**

We first included all PLHIV followed in the IeDEA West Africa cohorts between January 2017 and June 2021, with a documented switch to DTG during 2019–2021 and in care ≥36 months at the day of switch. Weight change was estimated using a two slope piecewise linear mixed model with change point at the switch date. Secondly, we emulated a sequence of target trials (ETT) based on the observational data, performing pooled logistic regression analysis to compare SWG occurrence between PLHIV who switched to DTG and those who did not.

**Results:**

We first included 6705 PLHIV from Burkina Faso, Côte d'Ivoire and Nigeria. Their median age at the time of switch was 48 years (IQR: 42–54) with a median follow‐up of 9 years (IQR: 6–12), 63% were female. Most patients switched from efavirenz (EFV)‐based ART (56.6%) and nevirapine (NVP)‐based ART (30.9%). The overall post‐switch annual average weight gain (AAWG) was significantly elevated at 3.07 kg/year [95% CI: 2.33–3.80] compared to the pre‐switch AWG which stood at 0.62 kg/year [95% CI: 0.36–0.88]. The post‐switch AWG was greater in patients previously on EFV and protease inhibitor (PI)‐based ART compared to those on NVP‐based ART. The pooled logistic regression analyses of a sequence of 24 ETT, including 9598 person‐trials, switching to DTG was significantly associated with an SWG (aOR = 2.54; 95% CI = 2.18–2.97).

**Conclusions:**

In West Africa, a 12‐month DTG exposure was associated with substantial weight gain, especially in PLHIV previously on EFV and PI‐based ARTs. Continuous weight monitoring and metabolic profiling is imperative in HIV cohorts to delineate the long‐term cardiometabolic impact of DTG as patients with, or at elevated risk for cardiovascular diseases might benefit from alternative ART regimens.

## 1 INTRODUCTION

Despite the widespread use of antiretroviral therapy (ART), the HIV epidemic remains a major global public health issue [1, 2]. The use of ART has presented many challenges including drug resistance, toxicity and side effects which collectively contribute to slow progress towards the UNAIDS third 95 target. Addressing these concerns, second‐generation integrase strand transfer inhibitors (INSTIs), dolutegravir (DTG), have demonstrated superior efficacy, improved tolerability and a higher barrier to the development of drug resistance [3, 4, 5, 6].

While advances in ART have significantly improved the health and life expectancy of people living with HIV (PLHIV) [7], the HIV care landscape is continuously changing and presenting new challenges. Indeed, the rising occurrence of non‐communicable diseases (NCDs), especially cardiometabolic diseases, is a major concern in the current era of long‐term HIV management [8, 9, 10, 11], especially as the risk of developing cardiometabolic diseases is greater in PLHIV who gain weight compared to persons without HIV [12, 13]. Weight gain is a well‐known and expected effect that often follows the initiation of ART primarily attributed to metabolic changes and a decrease in HIV‐induced inflammation [14, 15, 16]. In this situation, weight gain can be interpreted as “return to health” effect, particularly in contexts where ART is initiated at a more advanced stage of HIV infection [17, 18]. However, in patients on stable ART, progression to overweight and obesity may predispose to NCDs such as cardiovascular disease and type 2 diabetes [13, 19–21], which contribute significantly to morbidity and mortality in this population.

In recent years, several studies have shown that ART‐experienced PLHIV often experience weight gain following a switch to INSTI‐based treatments, especially DTG [22, 23, 24, 25, 26, 27]. This weight gain could be partly explained by the weight‐suppressive effects of previously used efavirenz (EFV) [28, 29]. However, evidence also suggests that DTG and other INSTIs may have a direct role in promoting weight gain [30]. Given its favourable profile, DTG was endorsed by the World Health Organization in 2018 as a preferred component of ART for HIV management, marking a significant shift in worldwide HIV care [31, 32]. Consequently, many sub‐Saharan African countries highly impacted by the HIV epidemic, including West African countries, have transitioned from predominantly non‐nucleoside reverse transcriptase inhibitor (NNRTI)‐based ART to DTG‐based ART [33, 34, 35, 36, 37, 38]. Despite the growing number of reports highlighting adverse metabolic effects of DTG in developed countries [25, 39], and more recently in a few African settings [26], available information from sub‐Saharan Africa remained limited. Although DTG's benefits are well‐established, it is critical to investigate its metabolic effects in diverse settings especially in sub‐Saharan Africa, as black race or being issued from some African countries has been identified as at increased risk of weight gain when initiating INSTIs [30].

The objectives of the present analyses were to evaluate changes in body weight in ART‐experienced PLHIV before and after they switch to a DTG‐based regimen and to assess if switching to a DTG‐based regimen was associated with a significant weight gain (SWG), defined as a ≥10% increase, compared to a reference population that remained on an NNRTI‐ or protease inhibitor (PI)‐based ART.

## 2 METHODS

### 2.1 Population and data sources

This study used routinely recorded, anonymized data from the International Epidemiology Databases to Evaluate AIDS West Africa (IeDEA‐WA) collaboration. The IeDEA‐WA, as part of the global IeDEA consortium, has been previously described elsewhere [40] (http://iedea‐wa.org). As part of the IeDEA‐WA collaboration, contributing HIV clinics share and merge data from PLHIV routine medical visits including medical records and laboratory test results. Research staff centrally extracted and validated all laboratory and clinical data, including anthropometric measures (weight) and medication start and stop dates, from the electronic medical record. For this analysis, data from the latest version of the IeDEA‐WA database (Version 8.0—04/13/2022) comprising nearly 50,000 adult PLHIV who ever initiated ART from seven HIV programmes in Benin, Burkina Faso, Cote d'Ivoire, Nigeria and Togo were used. Due to the unavailability of weight measures in four cohorts, we have restricted this analysis to three sites in three countries: The National Blood Transfusion Center (CNTSCI) in Abidjan, Côte d'Ivoire, the Souro Sanou University Hospital Center in Bobo‐Dioulasso, Burkina Faso and the National Institute of Medical Research in Lagos, Nigeria. These programmes contributed data collected from January 2017 to the database's closure in June 2021. Weight was collected and recorded in kilograms (kg) during routine study visits using a single measurement on a mechanical scale.

### 2.2 Study design and population

We conducted a cohort analysis using two different approaches.

The first approach (A1) aimed to evaluate weight changes before and after switching to a DTG‐based regimen. This part of the study included all ART‐experience PLHIV, aged 18 years or older, who were receiving care in one of the participating HIV clinics, and had switched to a DTG‐based regimen between 01/01/2019 and 02/01/2022. Participants were required to have been on ART for a minimum of 3 years prior to the DTG switch, with at least one recorded weight measurement in either the 24‐month period before the switch or the 12‐month period after the switch. The period of study ranged from 24 months before the switch (inclusion date) to 12 months thereafter.

The second approach (A2) aimed to assess the association between switching to DTG and SWG. This part of the study involved ART‐experienced PLHIV on ART for a minimum of 12 months at the introduction of DTG in the site. Participants were categorized into two groups: the switch group, comprising participants who had switch to DTG, and the reference group, those who did not. Two weight measurements were required for all participants. For the switch group, the first weight measurement considered was the one closest to the switch date within a ± 3‐month window, and the second, 12 months later within a similar interval. For the reference group, the baseline weight measurement considered was the first from the DTG introduction date, and the second, 12 months afterward within a ± 3‐month window. Pregnant women were excluded from both analyses.

### 2.3 Outcomes and covariate

Two primary outcomes were assessed in this study: the annual average weight gain (AAWG) before and after the switch in the first part of the analysis (A1), and the occurrence of SWG, defined as an increase in weight of 10% or more [14, 41], over a 12‐month period in the second part of the analysis (A2). Considered covariates included in both analysis, sex assigned at birth (sex), age categories (<30, 30–50 and >50 years), ART regimen at baseline (categorized into EFV‐based, nevirapine [NVP]‐based and PI‐based regimens plus two NRTIs), body mass index (BMI) classes (<18.5, [18.5–25], [25–30] and >30) and contributing HIV clinic. CD4 count (<350, [350–500] and ≥500 cells/mm^3^), viral load (VL) suppression defined as (yes if VL≤1000 copies/ml and no if ≥1000 copies/ml), duration on ART (in years) were included only in A2. VL suppression, CD4 count and BMI were the closest measure to baseline within a window period of ±3 months.

### 2.4 Statistical analysis

#### 2.4.1 Piecewise linear mixed modelling of repeated weight measurements

In our first analysis, weight change trajectories over the study period were modelled using piecewise linear mixed models (LMMs). We included all weight measurements within the study period. Change point was fixed at the date of the switch to estimate the AAWG before and after the switch to DTG‐based ART. We modelled piecewise (with two slopes) LMMs without covariates, testing progressively the contribution of random effects on the intercept and each of the two slopes. Random effects also accounted for the correlation of repeated measurements within each subject. Unstructured and diagonal covariance matrices were also compared. An LMM with random intercept and random slopes and with unstructured covariance was selected through the smallest Akaike information criterion. Univariable LMMs were used to assess covariate effects, and a manual backwards selection method was used to select significant covariates in a multivariable LMM. Covariates retained in the final model included sex, age classes, BMI at baseline, ART regimen prior to DTG switch and study site. To assess the adequacy of the model, residual homoscedasticity and normality were checked graphically. The average weight estimates in kg per year were plotted and stratified by ART regimen, sex and age classes.

#### 2.4.2 Emulated target trial with inverse probability of treatment weighting

We used a target trial emulation approach consisting of two steps: specifying the protocol of the target trial and emulating the target trial using observational data.

Participants eligible for this analysis are 18 years or older, followed in one of the three participating clinic sites for at least 1 year, being under non‐DTG ART and attending a clinical follow‐up visit during DTG transition from the start of the DTG rollout in each site from January 2019 to December 2020. Considered treatment strategies were: (i) initiating (i.e. switching to) an ART regimen containing DTG (individuals assigned to this strategy will be referred to as initiators) versus (ii) staying on a non‐DTG ART regimen. Eligible individuals would be randomly assigned to a strategy and would be aware of their assignment. Each participant would be followed up for 1 year, from assignment (time zero). The outcome of interest would be an SWG defined as a ≥10% increase over a 12‐month period. The causal contrasts of interest are the intention‐to‐treat (ITT) effect and the per‐protocol (PP) effect. The ITT analysis estimates 1‐year risk of SWG under each treatment strategy and compares them using a logistic regression model for the annual risk of event of SWG which included the treatment group as a covariate. The PP analysis is similar to the ITT analysis except that, participants are excluded if they did not complete the 1‐year weight measurement.

We emulated the target trials using observational data from the three sites of the IeDEA IeDEA‐WA collaboration previously mentioned. We identified eligible individuals in January 2019, and assigned them to the treatment strategy that was compatible with their data. This process was repeated until December 2020, emulating a sequence of 24 target trials with varying time zero. To emulate a randomized assignment, inverse probability of treatment weighting (IPTW) was performed to balance differences in baseline characteristics between the two treatment groups. We first estimated a propensity score (PS) for everyone as the predicted probability of switch compared to non‐switch from a multivariable logistic regression including sex, age classes, ART regimen, duration on ART, HIV VL suppression, BMI classes and monthly time period variable. We then estimated stabilized PS weight (SPSW) from the predicted PS and the marginal probability of receiving the treatment (*n*). The SPSW was *n*/PS if the participant has switched and (1–*n*)/(1–PS) if not. The causal contrast of interest is PP effect. The PP analysis estimates 1‐year risk of SWG under each treatment strategy and compares them using a pooled logistic regression model, among participants who completed a 1‐year weight measurement (± 3 months).

All statistical analyses were conducted using SAS version 9.4 software (SAS Institute Inc., North Carolina).

### 2.5 Ethical approval

The IeDEA‐WA Collaboration obtained authorization from the Ethics committee “Comite de Protection des Personnes Sud‐Ouest et Outre‐mer III” in Bordeaux, France (IRB00012788) to collect, merge and analyse de‐identified data from involved HIV clinics in West Africa. Moreover, each participating site obtained authorization from its National Ethics committee (Cote d'Ivoire: IRB00009111; Burkina Faso: IRB00004738; Nigeria [NIMR]: IRB00003224). Written informed consent requirements were deferred to the local institutional review boards. The analysis only used de‐identified data collected from routine clinical care.

## 3 RESULTS

### 3.1 Studied populations

Across the three contributing HIV clinics, 15,000 PLHIV have been monitored since the introduction of DTG, of which 13,909 patients were already on ART. Among these PLHIV, 7957 subsequently switched to a DTG‐based regimen and 5962 did not switch by the database closure date (Figure 1). Of those who switched, a total of 6705 participants were included in our primary analysis after excluding PLHIV who had less than 3 years of follow‐up by the date of the switch (*N* = 734), those without documented weight measurement (*N* = 32), women who had become pregnant at least once during the study period (*N* = 420) and all other patients for whom the ART regimen was missing or not clearly defined (*N* = 54).

Figure 1(a) Flow diagram of the selection of the studied populations (Analysis 1). (b) Selection of individuals for the emulation of a target trial, 2019–2020. ART, antiretroviral therapy; DTG, dolutegravir.*Non‐initiators of DTG could be included as initiators in subsequent trials if they initiated DTG and still met the eligibility criteria or several times as non‐initiators if they did not initiate DTG but still met the eligibility criteria.

**Figure:**
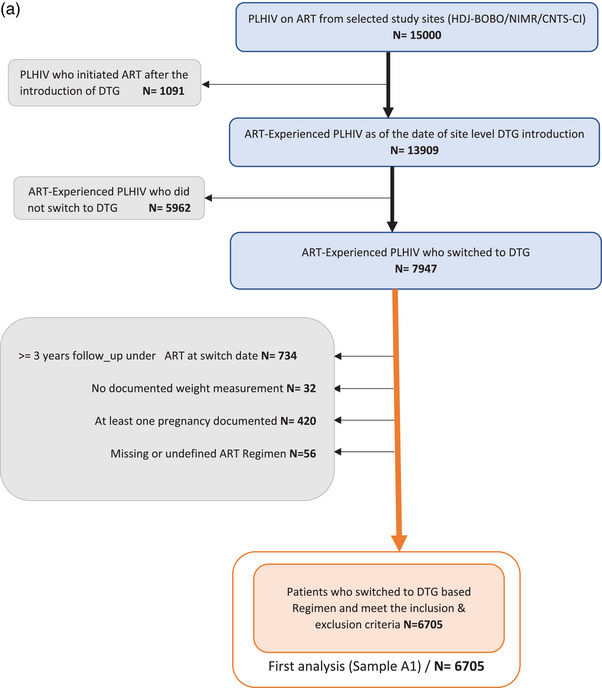

**Figure:**
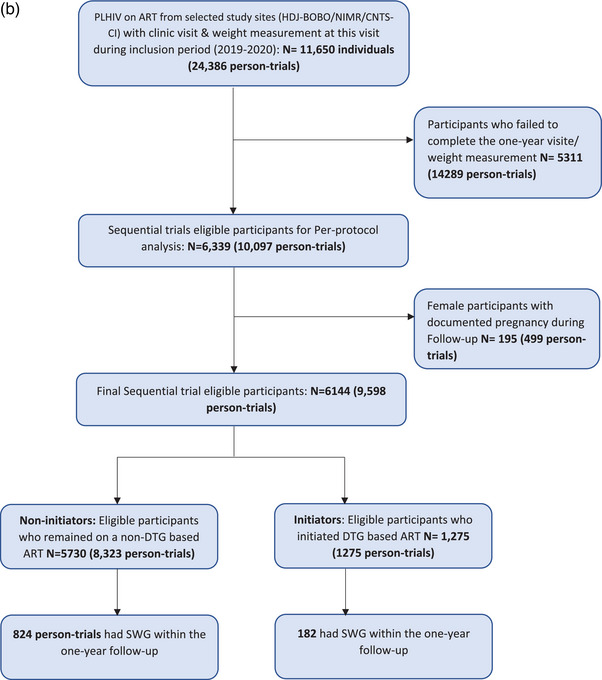

**Figure 2:**
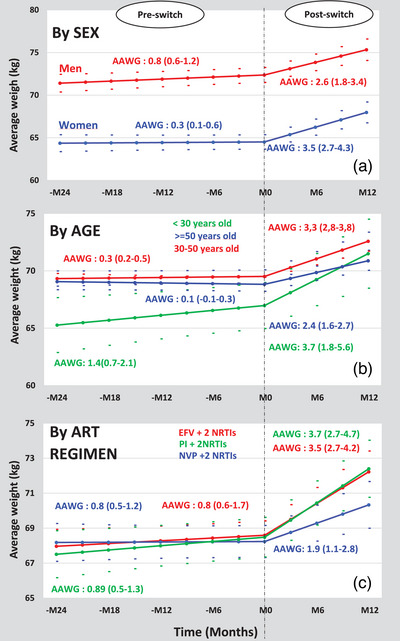
Weight evolution from month‐24 pre‐switch to month‐12 post‐switch to DTG according to sex, age and ART regimen pre‐switch. AAWG, annual average weight gain (in kg/year); EFV, efavirenz; NRTIs, nucleoside reverse transcriptase inhibitors; NVP, nevirapine; PI, protease inhibitor.

For the second analysis, of the 9598 person‐trials included in the PP analysis, 1275 have switched to a DTG‐based ART (initiators) and 8323 did not (non‐initiators) (Figure 2). Our selection process involved excluding participants who failed to complete the 1‐year visit and/or weight measurement (14,283 person‐trials) and women who experienced pregnancies during the period including 1 year before the start of follow‐up to the end of follow‐up (499 person‐trials).

### 3.2 Weight changes before and after switching to DTG

Participants were predominantly female (63%). The median age at inclusion was 48 years (IQR: 42–54). Median follow‐up since ART initiation was 9 years (IQR: 6–12). An HIV VL at the time of DTG switch was documented in 66.2% of participants and among them, 277 (6.2%) were detectable. Most of these participants had transitioned from EFV‐based (56.6%) and NVP‐based (30.9%) ART. The prevalence of overweight (BMI = [25–30]) and obesity (BMI≥30) were 24.7% and 17.2%, respectively, while BMI was missing in 22.1% of participants (Table 1).

**Table 1:** Participants’ characteristics at switch for assessing weight changes before and after the switch to DTG‐based ART (*N* = 6705)

	*n*	%
**Age in years (median) (IQR)**	48 (42–54)	
**Follow‐up since ART initiation (median) (IQR)**	9 (6–12)	
**Sex assigned at birth**		
Male	2473	36.88
Female	4232	63.12
**Age classes**		
<30 years	122	1.82
30–50 years	4322	64.46
> 50 years	2261	33.72
**CD4 count cells/mm^3^**		
<350	821	12.24
350–500	1130	16.85
>500	3653	54.48
Missing	1101	16.42
**Viral load suppression (<1000 copies/ml)**		
Yes	4162	62.07
No	277	4.13
Missing	2266	33.80
**Pre‐switch ART regimen**		
2NRTIs+EFV	3797	56.63
2NRTIs+NVP	2076	30.96
2NRTIs+PI	832	12.41
**BMI at switch**		
Underweight (BMI <18.5)	226	3.37
Normal (BMI = [18.5–25])	2186	32.60
Overweight (BMI = [25–30])	1656	24.70
Obese (BMI ≥30)	1153	17.20
Unavailable	1484	22.13
**Study site**		
HDJ‐BOBO	1340	19.99
CNTSCI	1623	24.21
NIMR	3742	55.81

*Note*: IeDEA West Africa collaboration. 20172022.

Abbreviations: CNTSCI, The National Blood Transfusion Center in Abidjan, Côte d'Ivoire; EFV, efavirenz; HDJ‐BOBO, Souro Sanou University Hospital Center in Bobo‐Dioulasso, Burkina Faso; NIMR, National Institute of Medical Research in Lagos, Nigeria; NRTIs, nucleoside reverse transcriptase inhibitor; NVP, nevirapine; PI, protease inhibitor.

Overall, baseline mean weight was 68.00 kg (95% CI: 67.37–69.06) and adjusted AAWG per year were estimated at 0.62 kg (95% CI: 0.36–0.88) and 3.07 kg (95% CI: 2.33–3.80) before and after the switch, respectively. Point estimates of AAWG varied by sex, age and previous ART regimens (Figure 2a–c).

Females, compared to males, had a lower baseline weight (65 vs. 71 kg) and a lower AAWG before the switch (0.36 vs. 0.88 kg), but a greater AAWG in the year following the switch (3.53 vs. 2.61 kg) (Table 2). This leads to a slightly reduced weight gap between men and women during the post‐switch period (Figure 2a). For age groups, we observed the following point estimates: before the switch, 1.42 kg (under 30 years), 0.34 kg (30–50 years) and a non‐significant estimate for those over 50 years; after the switch, 3.74 kg (under 30 years), 3.31 kg (30–50 years) and 2.14 kg (over 50 years). Regarding previous ART regimens, point estimates for pre‐switch AAWG were 0.42 kg (NVP‐based), 0.56 kg (EFV‐based) and 1.89 kg (PI‐based). Post‐switch AAWG point estimates were 1.97 kg (from NVP), 3.5 kg (from EFV) and 3.73 kg (from PI) (Figure 2c).

**Table 2:** Predicted baseline weight and annualized average weight gain before and after the switch to DTG‐based regimen from piecewise LMM (two slope)

		Adjusted average weight gain (kg per year)	
Variable at the time of switch	Adjusted baseline weight (kg) mean (95% CI)	Before the switch mean (95% CI)	After the switch mean (95% CI)
Overall	68.22(67.37; 69.06)	0.62 (0.36; 0.88)	3.07 (2.33; 3.80)
**Sex assigned at birth**			
Male	71.09 (70.17; 72.01)	0.88 (0.60; 1.17)	2.61(1.81; 3.40)
Female	65.35 (64.47; 66.22)	0.36 (0.09; 0.62)	3.53 (2.76; 4.29)
**Age classes**			
<30 years	65.04 (62.89; 67.19)	1.42 (0.74; 2.11)	3.74 (1.84; 5.65)
30–50 years	70.10 (69.50; 70.69)	0.34 (0.17; 0.50)	3.31 (2.79; 3.84)
>50 years	69.51 (68.86; 70.17)	0.10 (−0.08; 0.29)	2.14 (1.57; 2.70)
**ART regimen**			
2NRTIs+EFV	68.30 (67.44; 69.17)	0.56 (0.29; 0.82)	3.50 (2.75; 4.26)
2NRTIs+ PI	67.87 (66.78; 68.95)	0.89 (0.54; 1.23)	3.73 (2.75; 4.70)
2NRTIs+ NVP	68.48 (67.48; 69.48)	0.42 (0.12; 0.73)	1.97 (1.10; 2.84)
**Follow‐up duration since ART initiation**			
≤5 years	68.88 (67.86; 69.90)	0.62 (0.36; 0.88)	3.64 (2.74; 4.54)
>5 years	67.55 (66.73; 68.38)		2.49(1.76; 3.22)
**BMI**			
Underweight (BMI <18.5)	51.42 (49.75; 53.10)	−1.54 (−2.06; −1.02)	5.86 (4.47; 7.25)
Normal (BMI = [18.5–25])	59.99 (59.11; 60.87)	0.38 (0.12; 0.65)	3.59 (2.82; 4.35)
Overweight (BMI = [25–30])	72.08 (71.13; 73.03)	1.20 (0.91; 1.49)	2.15 (1.31; 2.98)
Obese (BMI ≥30)	87.23 (86.18; 88.28)	2.14 (1.82; 2.46)	1.03 (0.11; 1.94)
Unavailable	70.36 (69.34; 71.38)	0.92 (0.57; 1.27)	2.71 (1.72; 3.71)
**Study site**			
CNTSCI	68.13 (67.17; 69.10)	0.92 (0.62; 1.23)	3.63 (2.79; 4.46)
NIMR	69.10 (68.19; 70.03)	0.30 (0.02; 0.58)	3.52 (2.73; 4.32)
HDJ‐BOBO	67.41 (66.37; 68.46)	0.64 (0.32; 0.96)	2.05 (1.11; 2.99)

*Note*: IeDEA West Africa collaboration. 2017–2022.

Abbreviations: CNTSCI, The National Blood Transfusion Center in Abidjan, Côte d'Ivoire; EFV, efavirenz; HDJ‐BOBO, Souro Sanou University Hospital Center in Bobo‐Dioulasso, Burkina Faso; NIMR, National Institute of Medical Research in Lagos, Nigeria; NRTIs, nucleoside reverse transcriptase inhibitor; NVP, nevirapine; PI, protease inhibitor.

### 3.3 Association between switching to DTG and SWG

Compared to the non‐initiators, the DTG initiators group had a higher proportion of participants with male sex (49.2% vs. 26.5%), over 50 years old (43.8% vs. 24.4%), on EFV‐based ART (71.1% vs. 58.8%), from the CNTSCI site (66.6% vs. 55.9%) with less proportion of unsuppressed baseline HIV VL (3.7% vs. 7.6%). The corresponding SMDs were 0.48, 0.41, 0.32, 0.61 and 0.17, respectively. Obesity and overweight accounted for 17.7% and 25.5% in the non‐initiators group versus 15.6% and 25.3% in the initiators group, respectively. After weighting, it was verified that the variables included in the estimation of the propensity score and used for stabilized IPTW were well balanced between the two groups, with SMDs of <0.10 (Table 3).

**Table 3:** Inclusion characteristics of the study population according to the switch to DTG before and after weighting (*N* = 3682)

	Before weighting (*N* = 9598 person‐trials)					After weighting (*N* = 9598 person‐trials)				
	Non‐initiators (*N* = 8,323)		Initiators (*N* = 1275)		SMDs	Non‐initiators		Initiators		SMDs
	*n*	%	*n*	%		*n*	%	*n*	%	
**Sex**					0.4816					−0.029
Male	2203	26.5	627	49.2		2453.89	29.51	359.135	28.21	
Female	6120	73.5	648	50.8		5862.01	70.49	914.13	71.79	
**Age**					0.4138					0.051
<30 years	373	4.5	28	2.2		347.13	4.17	61.4238	4.82	
30–50 years	5920	71.1	688	54.0		5726.75	68.87	872.19	68.50	
> 50 years	2030	24.4	559	43.8		2242.02	26.96	339.65	26.68	
**Viral load suppression (<1000 copies/ml)**					0.1774					0.099
Yes	4925	59.2	750	58.8		4928.65	59.27	677.46	53.21	
No	632	7.6	47	3.7		589.51	7.09	155.89	12.24	
Missing	2766	33.2	478	37.5		2797.74	33.64	439.91	34.55	
**CD4 cells count/mm^3^**					0.1254					0.087
<350	1183	14.2	135	10.6		1145.65	13.78	168.74	13.25	
350—500	1162	14.0	152	11.9		1142.56	13.74	165.43	12.99	
>500	3584	43.1	561	44.0		3595.82	43.24	523.224	42.09	
Missing	2394	28.7	427	33.5		2431.88	29.24	415.87	32.70	
**ART regimen at baseline**					0.3227					0.001
2NRTIs+EFV	4893	58.8	907	71.1		5021.53	60.38	768.85	60.38	
2NRTIs+NVP	1060	12.7	184	14.4		1076.55	12.95	165.80	13.02	
2NRTIs+PI	2370	28.5	184	14.4		2217.81	26.67	338.61	26.59	
**BMI at baseline**					0.1682					0.072
Underweight (BMI <18.5)	369	4.4	90	5.2		374.77	4.51	87.29	5.70	
Normal (BMI = [18.5–25])	3265	39.2	861	45.5		3329.35	40.04	777.59	40.20	
Overweight (BMI = [25–30])	2124	25.5	539	25.3		2124.56	25.55	484.48	25.93	
Obese (BMI ≥30)	1474	17.7	380	15.6		1448.72	17.42	384.51	15.39	
Unavailable	1091	13.1	96	8.4		1038.5	12.50	117.04	12.80	
**Study site**					0.6146					0.081
HDJ‐BOBO	1900	22.8	215	45.8		1833.08	22.04	316.89	24.90	
CNTSCI	3238	38.9	849	20.9		3538.02	42.55	542.48	42.61	
NIMR	3185	38.3	211	33.3		2944.81	35.41	413.89	32.51	

*Note*: IeDEA West Africa collaboration. 2017–2022.

Abbreviations: CNTSCI, The National Blood Transfusion Center in Abidjan, Côte d'Ivoire; EFV, efavirenz; HDJ‐BOBO, Souro Sanou University Hospital Center in Bobo‐Dioulasso, Burkina Faso; NIMR, National Institute of Medical Research in Lagos, Nigeria; NRTIs, nucleoside reverse transcriptase inhibitor; NVP, nevirapine; PI, protease inhibitor; SMDs, standardized mean differences.

Of the 9598 person‐trials, initiators were more likely to experience SWG over the 12‐month follow‐up period, 182 (14.27%), compared to the non‐initiators, 824 (9.90%).

In a PP analysis of the sequence of 24 monthly ETT, using a pooled logistic regression model adjusted on monthly time, with stabilized IPTW to balance baseline characteristics, initiating DTG among ART‐experienced PLHIV were at increased risk of SWG (aOR = 2.54; 95% CI = 2.18–2.97).

## 4 DISCUSSION

In this observational study, we assessed the impact of switching to DTG on weight gain in ART‐experienced PLHIV prospectively monitored in a context of programmatic transition towards DTG‐based regimens in West Africa. We found a greater AAWG of 3.1 kg in the 12 months following the switch to DTG, compared with an AAWG of 0.6 kg in the 2 years prior to the switch. Our findings also highlighted a greater weight gain among patients switching from EFV and PI‐based ART compared to NVP‐based ART.

In line with previously published literature on switch to DTG in resource‐rich settings [22, 24, 25, 27] and more recently by the AFRICOS study group [26] and by Brennan et al. [23] in sub‐Saharan Africa, we confirmed a substantial overall AAWG following the switch to DTG. In contrast, Burns et al. in the United Kingdom [39], Guaraldi et al. in Italy [42] and Hickey et al. in rural Kenya [43] found no or limited weight gain after the switch to DTG. The difference in findings may partly be attributed to population characteristics, with the British cohort having a high BMI at the time of the switch, while the Italian cohort was a geriatric one and both cohorts were predominantly males. Prevalent food insecurity in the rural Kenyan cohort suggests that dietary practices could influence DTG‐related weight gain mechanisms.

In previously published studies on ART‐experienced PLHIV switching to INSTIs and particularly to DTG, switching from EFV was mostly associated with greater weight gain [22, 23, 25, 26]. Our study includes patients mainly on EFV‐based ART as prior ART regimen strongly supports these findings. One potential mechanism suggests that the weight gain associated with the switch to DTG might result from the elimination of the weight‐suppressive effect inherent to the preceding ART regimen [28], predominantly EFV in sub‐Saharan Africa [44]. Other studies have proposed that DTG might exert a specific effect on weight gain through the activation of the melanocortin‐4 receptor and could directly enhance adipogenesis and insulin resistance [45]. In contrast to reports showing no or limited weight gain following the switch from a PI‐based regimen to DTG, [27, 46], PI‐based ART at the time of the switch was associated with increased AAWG and SWG in our study. However, the post hoc analysis of the 96‐Week NEAT‐022 Randomized Trial found that switching from PI to DTG in PLHIV with high cardiovascular risk led to modest weight gain limited to the first 48 weeks. Lake et al. had specifically shown a significant increase in annualized weight gain when switching from a PI to DTG [25].

Prior studies have also reported consistent findings on the association between female sex (compared to male) and weight gain following the switch to DTG [25, 26, 43, 47]. Although the mechanism underlying sex difference remains unclear, mid‐life hormonal changes in women could contribute to the differential in weight gain [48, 49].

A few studies have monitored weight changes over a period exceeding a 12‐month follow‐up. Findings suggest that beyond 2 years, the weight gain linked to DTG switch is no longer significant [24]. Due to limited follow‐up duration, evidence describing the metabolic effects or other implications of weight gain with INSTIs is still scarce. Some studies show an increased risk of hypertension or diabetes, while others have highlighted an increased risk of dyslipidaemia [50, 51, 52, 53]. Conversely, others found no clinical implications of this weight gain associated with INSTIs [54, 55]. Further long‐term studies are needed to consistently address these questions.

Some limitations need to be considered when interpreting the results of this study. First, it is important to note that the observed differences in AAWG by sex, age and prior ART regimens are descriptive only and should not be interpreted as causal due to methodological limitations, including the risk of Table 2 fallacy. Second, missing weight data, leading to participant exclusion in the per‐protocol analysis of the second analysis, could limit generalizability. However, the emulated target trial approach helps mitigate selection bias. Third, as the study took place during the programmatic DTG transition, there was an inherent difference between switchers and non‐switchers. We addressed this potential selection bias using the participants’ own pre‐switch weight trajectory among switchers and IPTW to balance confounding factors. In addition, important unmeasured factors such as lifestyle‐related elements (e.g. dietary habits, physical activity), adherence to ART or socio‐psychological conditions that might influence weight fluctuations, could potentially affect our results, especially since these factors were not considered in IPTW. Finally, our 10% weight increase threshold for SWG, while widely used, lacks consensus; notably, a >5% gain has been linked to insulin resistance in PLHIV [24]. However, the absence of a universal agreement on what constitutes a clinically meaningful weight gain threshold could affect the implications of our findings.

## 5 CONCLUSIONS

Our study provides evidence that switching to DTG in ART‐experienced PLHIV is associated with a significant AAWG, especially when transitioning from EFV and PI‐based ART regimens in West Africa. Our results underscore the substantial weight gain among individuals switching from EFV‐based ART, potentially due to the cessation of weight‐suppressive effects of the previous regimen or direct effects of DTG on weight. Despite the use of an emulated target trial approach, ideally, a randomized controlled trial would provide the most robust evidence to support the conclusions presented in this manuscript. Questions remain about the long‐term metabolic implications of this weight gain. Given the potential impact on hypertension, diabetes and other cardiovascular events, ongoing research into the long‐term effects of DTG‐related weight gain requires a continuing, rigorous weight and metabolic profiling in HIV cohorts.

## COMPETING INTERESTS

The authors declare no competing interests.

## AUTHORS’ CONTRIBUTIONS

TT and AJ conceived the study concept. TT and KM were in charge of the data management. TT performed the statistical analysis and wrote the first draft of the manuscript with significant inputs from AJ. KM, AP, AM, CDL, OE, DKE, IO and AJ reviewed, provided critical inputs and approved the final version of the manuscript.

## FUNDING

Research reported in this publication is supported by the U.S. National Institutes of Health, National Institute of Allergy and Infectious Diseases, the Eunice Kennedy Shriver National Institute of Child Health & Human Development, the National Heart, Lung, and Blood Institute, the National Institute of Diabetes and Digestive and Kidney Diseases, the National Institute on Drug Abuse, the National Institute on Alcohol Abuse and Alcoholism, the Fogarty International Center and the National Cancer Institute, under Award Number U01AI069919.

## DISCLAIMER

This work is solely the responsibility of the authors and does not necessarily represent the official views of any of the institutions mentioned.

## Data Availability

The data that support the findings of this study are available from the corresponding author upon reasonable request.
